# Radiation Induced Surface Modification of Nanoparticles and Their Dispersion in the Polymer Matrix

**DOI:** 10.3390/nano10112237

**Published:** 2020-11-11

**Authors:** Zhiang Fu, Xiaoying Gu, Lingmin Hu, Yongjin Li, Jingye Li

**Affiliations:** 1 CAS Center for Excellence on TMSR Energy System, Shanghai Institute of Applied Physics, Chinese Academy of Sciences, No. 2019, Jialuo Road, Jiading District, Shanghai 201800, China; fuzhiang@sinap.ac.cn; 2 College of Material, Chemistry and Chemical Engineering, Hangzhou Normal University, No. 16 Xuelin Rd., Hangzhou 310036, China; 13750803291@163.com (X.G.); hulingmin0201@126.com (L.H.); 3 University of Chinese Academy of Sciences, Beijing 100049, China

**Keywords:** radiation grafting, nanoparticles, PVDF, silica, dispersion

## Abstract

Polymer grafted inorganic nanoparticles attract significant attention, but pose challenges because of the complexity. In this work, a facile strategy to the graft polymer onto the surface of nanoparticles have been introduced. The vinyl functionalized SiO_2_ nanoparticles (NPs) were first prepared by the surface modification of the unmodified SiO_2_ using γ-methacryloxy propyl-trimethoxylsilane. The NPs were then mixed with polyvinylidene fluoride (PVDF), which was followed by the Co-60 Gamma radiation at room temperature. PVDF molecular chains were chemically grafted onto the surface of SiO_2_ nanoparticles by the linking of the double bond on the NPs. The graft ratio of PVDF on SiO_2_ NPs surface can be precisely controlled by adjusting the absorbed dose and reactant feed ratio (maximum graft ratio was 31.3 wt%). The strategy is simple and it should be applied to the surface modification of many other nanoparticles. The prepared PVDF-grafted SiO_2_ NPs were then dispersed in the PVDF matrix to make the nanocomposites. It was found that the modified NPs can be precisely dispersed into the PVDF matrix, as compared with pristine silica. The filling content of modifications SiO_2_ NPs on the PVDF nanocomposites is almost doubled than the pristine SiO_2_ counterpart. Accordingly, the mechanical property of the nanocomposites is significantly improved.

## 1. Introduction

Organic–inorganic hybrid nanocomposites have attracted extensive interest because of their excellent comprehensive properties through the synergism of an inorganic and organic counterpart [1,2,3]. Unfortunately, simply dispersing inorganic nanoparticles in an organic (polymer) matrix is usually prone to phase segregation [4]. In order to achieve more efficient overall performance between inorganic and organic moieties, physical or chemical surface modification of the nanoparticles with organic ligands is necessary [2,5]. For physical methods, the physisorption of polymer chains onto particles surfaces with van der Waals interactions or hydrogen bonds were improved [6,7,8,9] while, for traditional chemical modifications, the enhancement of the synergistic effect was to create a covalent bond between macromolecules and the particles’ surface by various grafting reactions, including “grafting through,” “grafting from,” and “grafting onto” [10,11,12,13,14]. However, the most common grafting reactions are generally focused on the reactive polymers and nanoparticles, which required both the polymer and nanoparticle surface to have good reactivity groups [15,16]. For weak reactivity components, various reaction groups have to have an additional modification, which needs a delicate operation, severe pre-treatment, and a costly/complex synthetic technique [17,18,19,20]. It is necessary to explore a more simple strategy to circumvent these problems to construct the nanohybrid effectively.

Recently, radiation grafting methods have been found to be one of the versatile means for the preparation of a range of nanohybrids with different functionalities [21,22]. This makes it possible to combine a functional component with polymers available in various physical forms. Radicals can be created on the polymer by radiation and react with the reactive group (typically vinyl group) of the desired counterpart. Therefore, the nanohybrid obtained by this method are advantageous in terms of preparation, tailored composition, and tuned the desired characteristics [23,24]. For instance, Wu et al. [25] have proposed an idea that produced titanium dioxide coatings on ultra-high molecular weight polyethylene (UHMWPE) fabric by a radiation-induced graft polymerization process. The thermal and ultraviolet resistance of UHMWPE fabric is improved. However, the current application of the radiation grafting strategy is mainly focused on the polymer functionalization. Few investigations have been carried out to surface modification of nanoparticles with the polymer by this process.

**Figure 1:**
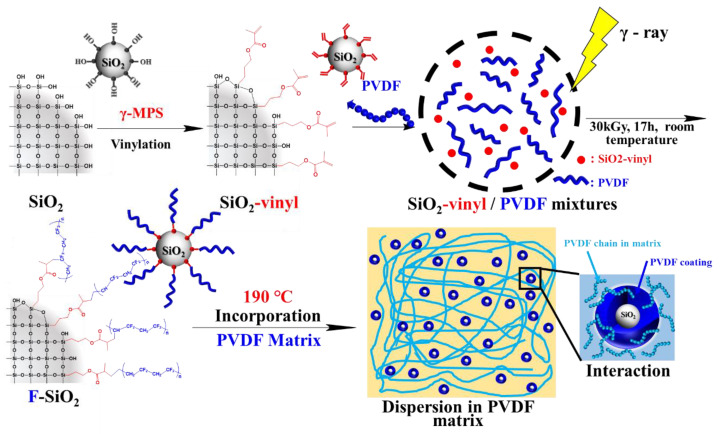
Schematic diagram of the surface modified silica nanoparticles with polyvinylidene fluoride (PVDF) coating by using radiation-induced graft polymerization under a wide temperature range and the distribution of F-SiO_2_ in the PVDF matrix.

In the present work, we propose a new strategy to surface modify the silica (SiO_2_) nanoparticles with polyvinylidene fluoride (PVDF) based on the “radiation grafting” process. As shown in Figure 1, the vinyl functionalized SiO_2_ NPs were first prepared by the surface modification of the unmodified SiO_2_ using γ-methacryloxy propyl-trimethoxylsilane. The PVDF moiety was then covalently bonded to the NPs via radicals reacting with vinyl groups on the silica surface under Co-60 Gamma radiation. The structure of functionalized NPs was extensively characterized, and the effect of the reaction parameters under radiation on silica binding PVDF was investigated. It should be noted that, due to the radiation grafting method, all reactions above were conducted under extremely mild conditions. PVDF can be grafted onto the surface of NP directly rather than traditional chemical modification that involves additional complex procedures. Meanwhile, the PVDF-grafted SiO_2_ NPs (F-SiO_2_) can be well dispersed into the PVDF matrix, as compared with pristine silica, since the PVDF chain on the silica surface improved the compatibility between nanoparticles and the matrix.

## 2. Materials and Methods

### 2.1. Materials

The poly(vinylidene fluoride) (PVDF, KF850) was purchased from Kureha Chemicals Japan (Ibaraki, Japan) and dried in vacuum at 80 °C for 24 h before use. Nanosilica with a mean size of 10 nm was purchased from Carbot bluestar Co. China (Jiujiang, China) and dried in vacuum at 200 °C for 12 h before use. Acetone (≥98.0%), Dimethyl sulfoxide (DMSO, ≥98.0%), N, N-Dimethylformamide (DMF, ≥99.5), Dimethylsulfoxide (DMSO, ≥99.5), and Ethanol absolute (≥99.8%) andγ-methacryloxy propyl-trimethoxyl silane (γ-MPS, ≥98.0%) were all purchased from a Sinopharm Chemical Reagent (Shanghai, China). All reactions were carried out under a nitrogen condition unless otherwise stated.

### 2.2. General Procedure for Exterior Functionalization of SiO_2_ with Vinyl Groups (SiO_2_-Vinyl)

SiO_2_-vinyl was prepared similarly to the method demonstrated in our previous work [26,27,28]. In addition, 5 g of silica NPs was charged into 250 mL DMF and the mixture was ultrasonicated for 15 min. Then, the suspension and γ-MPS (7.5 mL, 3 vol%) were transferred into a three-necked flask equipped with an N_2_ inlet and refluxed at 110 °C for 8 h. The reaction mixture was centrifuged and washed thoroughly with acetone and ethanol to remove the unreacted γ-MPS, respectively, and SiO_2_-vinyl was dried under vacuum at 65 °C for 24 h.

### 2.3. General Procedure for Grafting PVDF Chains onto the Exterior Surface of SiO_2_-Vinyl by the “Radiation Grafting” Method (F-SiO_2_)

F-SiO_2_ was available by grafting the PVDF chain onto SiO_2_-vinyl through the radiation grafting method in the presence of Co-60 gamma-ray irradiation. A typical procedure was as follows: SiO_2_-vinyl (1 g) was first dissolved into 50 mL of DMF and ultrasonicated for 5 min, respectively. Then, 0.1–2 g PVDF was incorporated to SiO_2_-vinyl by solution blending, respectively. The PVDF was grafted onto SiO_2_-vinyl by exposing the PVDF/SiO_2_-vinyl mixture to gamma-ray at 30–70 kGy for 17 h under room temperature. All samples were centrifuged and washed thoroughly with DMSO and DMF to remove the engrafted PVDF. The washing procedure was repeated three times and F-SiO_2_ was dried under vacuum at 100 °C for 24 h.

### 2.4. Preparation of Blending Materials

PVDF was dried in a vacuum oven at 80 °C for a minimum of 24 h prior to compounding. The various surface modified silica was incorporated into the PVDF matrix through a simple melt-blending method directly in a Haake Polylab QC (Thermo Fisher Scientific, Germany) mixer at 190 °C for 10 min. The screw speed of the mixing was 50 rpm/min. The nanocomposites were then compression-molded by hot pressing at 20 MPa and 190 °C for 15 min and followed by quenching to room temperature.

### 2.5. Characterizations

*Fourier Transform Infrared Spectroscopy (FTIR).* FTIR spectra were acquired using a VERTEX 70 V spectrometer (Bruker, Germany) at room temperature under vacuum. Samples were ginned and compressed into KBr flake over the range of 4000–400 cm^−1^ at a resolution of 2 cm^−1^ with a minimum of 64 scans added to obtain each spectrum. 

*Nuclear magnetic resonance (NMR)*. The chemical structure of the modified silica was analyzed by ^1^H-NMR and ^13^C-NMR using an AVANCE III 500 MHz spectrometer (Bruker, Germany). Samples were dissolved in DMSO. The operating frequency was 500 MHz.

*Thermal analysis.* Thermogravimetric analysis (TGA) curves were carried out on a TGA Q500 (TA Instrument, New Castle, DE, USA-) in nitrogen and air atmosphere from 40 °C to 650 °C. The heating rate was 10 °C/min. The content of grafted PVDF and vinyl groups on the silica surface was calculated by the equation in Formula S1 in the support information. The differential scanning calorimeter (DSC) curves were carried out on a DSC Q2000 (TA Instrument, New Castle, DE, USA) in nitrogen atmosphere from −50 °C to 220 °C. The heating rate was 10 °C/min. Dynamic mechanical analysis (DMA) curves were performed on a DMA—Q800 (TA Instruments, New Castle, DE, USA) in the tension mode and under a nitrogen atmosphere from −50 to 220 °C. The frequency was 3 Hz and the heating rate was 10 °C/min. The samples were tailored to dimensions of 8 mm × 6.30 mm × 0.50 mm in length, width, and thickness, respectively.

*Rheological testing.* Oscillatory rheological characterizations were carried out on a physical rheometer MCR301 (Anton Paar Instrument, Graz, Austria) in nitrogen at 200 °C. The diameters of the parallel plates were 25 mm and the gap between the two plates was 1 mm. The strain amplitude was set to be 1%. The frequencies used in this system ranged from 0.01 to 25 rad/s. 

*Mechanical testing.* Tensile tests were performed with an Instron 5966 universal testing machine (Instron, USA) at a crosshead speed of 10 mm/min. Samples were punched into tensile specimens with a standard dumbbell shape and aged for 24 h at room temperature prior to measurements. 

*Electron microscopy.* Transmission electron microscopy (TEM, Hitachi HT-7700, Tokyo, Japan) was used to observe the microstructure of various surface modified silica operating at an accelerating voltage of 100 kV. The specimens were first dissolved in DMF and dropped on the formvar stabilized with carbon support films, which dried under vacuum at 60 °C for 8 h. The dispersion of various surface modified silica in the PVDF matrix were analyzed by scanning electron microscopy (SEM, Hitachi, S-4800, Tokyo, Japan) and Atomic Force Microscope (AFM, Nanocute, E-Sweep, Tokyo, Japan). Energy dispersive X-ray (EDX) spectra and elemental mapping of the samples were performed on a Zeiss-4800 (ZEISS, Oberkochen, Germany). The specimens were fractured by immersion in liquid nitrogen for 5 min and then sputter-coated with Au prior to analysis.

## 3. Results and Discussion

### 3.1. Preparation of F-SiO_2_: Functionalization of SiO_2_

**Figure 2:**
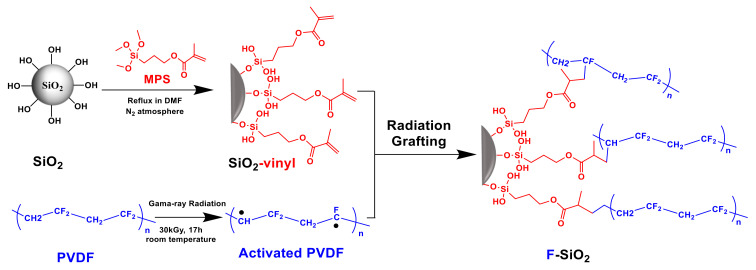
Illustrative synthesis of F-SiO_2_ through a “radiation-induced graft polymerization” method.

The fabrication procedure of the surface modified silica NPs is illustrated in Figure 2. The functionalization of SiO_2_ NPs with a PVDF chain (F-SiO_2_) was synthesized by sequential immobilization of reactive vinyl groups and PVDF chains onto the exterior surface of SiO_2_ using a silane coupling agent and commercially available PVDF, respectively. First, the pristine SiO_2_ was functionalized with vinyl groups by chemical modification using γ-methacryloxy propyl-trimethoxylsilane to yield reactive SiO_2_ (Vinyl-SiO_2_). Then, a small amount of PVDF was added and directly rooted on the SiO_2_ surface by gamma-radiation, which created radicals to initiate the efficient reaction with the vinyl group during the conditions and formed side chain grafts [23]. The newly developed process has the following advantages: the graft reactions between SiO_2_-vinyl and PVDF can be initiated over a wide temperature range including sub-ambient levels, making it a reproducible preparation of hybrid nanoparticles for industrial production.

**Figure 3:**
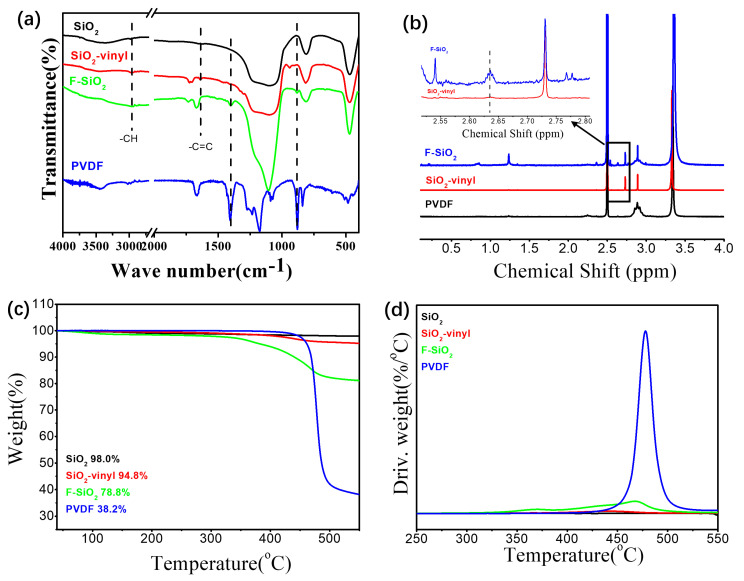
FT-IR (**a**), ^1^H-NMR (**b**), TGA (**c**), and DTG (**d**) curves of the SiO_2_, SiO_2_-vinyl, F-SiO_2_, and polyvinylidene fluoride (PVDF).

Figure 3 showed the FTIR, H^1^-NMR, TGA, and DTG curves of the silica NPs with various surface natures. As shown in Figure 3a, the strong and wide bands at 817 cm^−1^ and 1080 cm^−1^ was the absorption due to the asymmetric bending vibration of Si–OH and asymmetric stretching vibration of Si–O–Si, respectively, which can be observed in all the samples of modification silica, confirming the existence of SiO_2_ in the hybrid nanoparticles. Compared with the pristine SiO_2_, the peak of Si–OH became weaker and the new peaks at 2810–3050 cm^−1^, 1715 cm^−1^, and 1640 cm^−1^ can be observed for the Vinyl-SiO_2_ samples. These peaks were assigned to the stretching vibration of –CH, C=O, and C=C from γ-MPS, respectively, indicating the occurrence of a reaction between silica hydroxyl of pristine SiO_2_ and methoxyl of γ-MPS. In addition, the characteristic absorption peaks of PVDF at 1408 cm^−1^ and 880 cm^−1^ were also observed in the F–SiO_2_ samples. Since the unreacted PVDF was removed by extensive washing with organic solvents, the spectrum of F-SiO_2_ means PVDF chains successfully grafted onto the silica surface. Furthermore, ^1^H-NMR (Figure 3b) also demonstrated the appearance of γ-MPS (2.63 ppm) and PVDF (2.85 ppm, 2.89 ppm, and 2.91 ppm) for F-SiO_2_. The similar results can be also observed in the ^13^C-NMR (Figure S1) [29]. It implied that the reaction of SiO_2_ with γ-MPS occurred and PVDF was successfully grafted onto the surface of silica NPs, confirming further the PVDF was successfully grafted onto the surface of silica NPs.

TGA was used to measure the effect of modification on thermal stability of silica NPs. The TGA and derivative thermogravimetric analysis (DTG) curves was given in Figure 3c,d. It is clear that the thermal degradation of pristine silica nearly did not occur and the degradation of silica was almost negligible. With the modification of γ-MPS on the silica surface, the thermal weight loss was reduced (from 98.0% to 94.8%) and shown a thermal degradation temperature at 440 °C. In comparison, the further incorporation of PVDF leaded to a significantly decrease in the thermal weight loss (about 35.9 wt% of the PVDF grafting ratio) and improved the thermal stability of silica NPs (from 440 °C to 467 °C), which was close to 477 °C of pristine PVDF. The detailed molecular parameters of the surface modified silica NPs were shown in Table S1. These data confirmed again that F-SiO_2_ was successfully prepared via the radiation grafting process.

**Figure 4:**
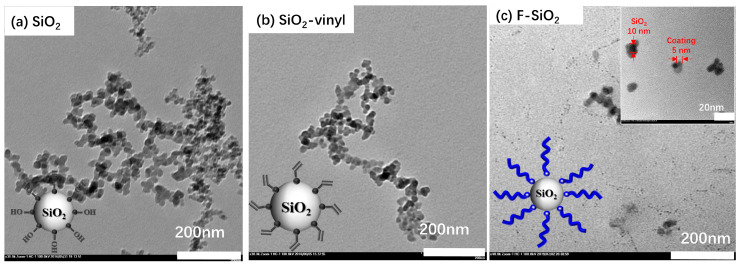
TEM image of the SiO_2_ (**a**), SiO_2_-vinyl (**b**), and F-SiO_2_ (**c**).

The same result was also clearly confirmed by TEM measurements. All the silica with an average diameter of 10 nm were used as model nanoparticles (Figure 4). Pristine SiO_2_ showed a typical agglomeration dropping cast onto a TEM grid (Figure 4a), which was contributed to the intense original characteristic of NPs. The modification silica with the vinyl group by silane coupling agents can remarkably improve the dispersibility of silica, while the NPs still show slight aggregation. While the gray corona of the γ-MPS on silica NPs surface was inconspicuous due to the small size and low molecular weight of silane coupling agent (Figure 3b). Compared with the pristine SiO_2_ and SiO_2_-vinyl, an explicit polymer coating layer (about 5-nm thickness) can be observed on the surface of the F-SiO_2_ after the radiation grafting of PVDF and the dispersion of the F-SiO2 was enhanced significantly. The size of F-SiO_2_ nanoparticles is about 5 nm based on observing TEM. The similar result was demonstrated in the X-ray diffraction (XRD) of the surface modification nanoparticles (Figure S2a,b). The original SiO_2_ showed a dominant XRD peak from amorphous structures of silica at 21.5°. With the increase of a modification degree, the peak was shifted to the lower wave number and the half-peak breadth was decreased due to the increase in nanoparticles size. To determine the crystal structures of F-SiO_2_ hybrid nanoparticles more accurately, the XRD Rietveld refinements [30] taken at room temperature were investigated by using the GSAS program (Figure S2c). It was found that the characteristic peaks of the α-phase crystal in the PVDF chain can be observed at 2θ = 19.4° in the F-SiO_2_ samples. This again means that the F-SiO_2_ was successfully prepared via the radiation grafting process and the effects of structure evolution of hybrid nanoparticles on crystallization behavior is still being studied.

### 3.2. Interface Modification Control of the PVDF Graft Ratio on Silica

**Figure 5:**
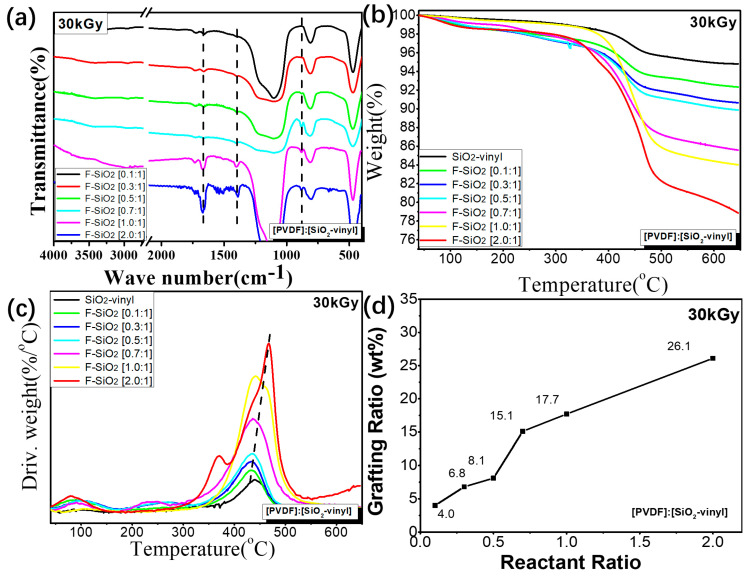
FT-IR (**a**), TGA (**b**), DTG (**c**) and the grafting ratio curves (**d**) of the F-SiO_2_ with a different [PVDF]/[SiO_2_-vinyl] reactant ratio under 30 kGy, 17 h Gamma-radiation condition.

To get further information on the control of PVDF graft ratio on silica by this strategy, FTIR and TGA of the F-SiO_2_ NPs with different reactant ratio was performed, and the results were shown in Figure 5. It can be clearly seen that, with the increasing feed ratio of the PVDF, the intensity of the PVDF characteristic peak (1408 cm^−1^ and 880 cm^−1^, Figure 5a) and the thermal weight loss of the organic component (Figure 5b) in the F-SiO_2_ samples were significantly enhanced under the same absorption dose and reaction time (30 kGy, 17 h). The maximum weight loss temperature of the F-SiO_2_ was gradually improved in the same time frame (from 433 °C to 467 °C, Figure 4c), suggesting that the thermal stability increased with the rise of the grafting degree of PVDF. These results were mainly attributed to the increase of the PVDF feed ratio, which can significantly improve the reaction opportunities between the vinyl group on the surface of nanoparticles and the PVDF radicals generated during a gamma-ray radiation process, leading to the grafting degree of PVDF on the surface of nanoparticles being controlled.

**Figure 6:**
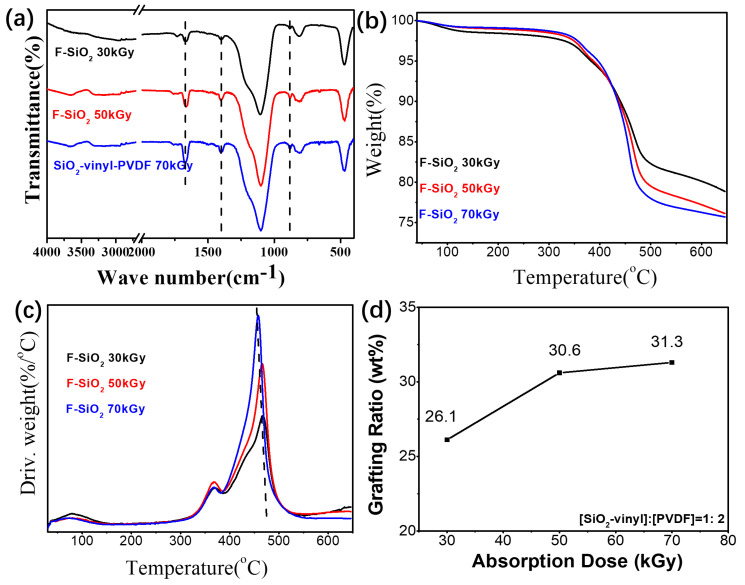
FT-IR (**a**), TGA (**b**), DTG (**c**) and the grafting ratio (**d**) curves of the F-SiO_2_ under a different absorbed dose and a 17-h Gamma-radiation condition. The reactant ratio of [SiO_2_-vinyl]:[PVDF] = 1:2.

On the other hand, increasing the absorption dose of the grafting reaction can achieve the same effect as the strategy of change in the feed ratio (Figure 6). The higher radiation dose can improve the production of radicals in PVDF chain to react with SiO_2_-vinyl, leading to the grafting degree of PVDF on the silica surface increasing (Figure 6a,b). However, compared with the 50 kGy radiation samples, the graft ratio of F–SiO_2_ with the 70 kGy absorbed dose was not significantly improved and a clear reduction in the thermal stability of the F-SiO_2_ was observed with the increase of the absorbed dose (Figure 4c). It indicated that the improvement of regulating the grafting degree by the absorption dose was limited and the degradation of PVDF will be dominant depending on the radiation conditions. A higher radiation dose not only leads to high-energy consumption but also impairs the molecular weight of PVDF, which influenced the thermal stability of the F–SiO_2_. Therefore, the maximum graft ratio of PVDF on silica NPs we used in the present work were prepared under the 30 kGy adsorption dose. Free radical formation together with little chain scission are the main concern. The detailed molecular parameters of the surface modified silica NPs with PVDF were shown in Table S2.

### 3.3. Dispersion Property of Modification Silica in the PVDF Matrix

**Figure 7:**
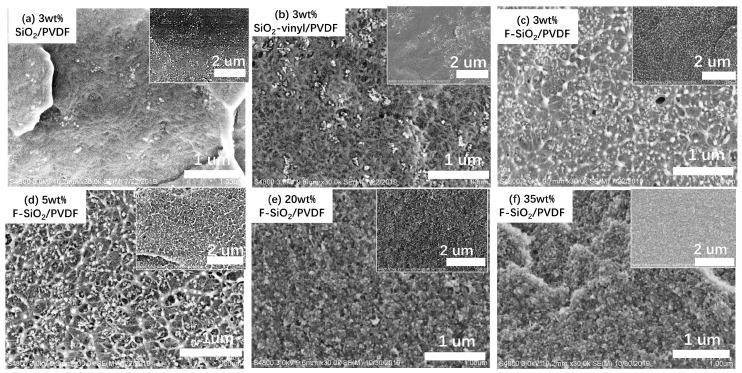
SEM images of the PVDF matrix incorporated with 3wt%, (**a**) pristine SiO_2_, (**b**) SiO_2_-vinyl, and (**c**) F-SiO_2_ nanoparticles, respectively. SEM images of PVDF matrix incorporated 5 wt% (**d**), 20 wt% (**e**), 35 wt%, and (**f**) F-SiO_2_ nanoparticles, respectively.

This allows a meaningful appraisal of the performance of surface modification silica by the radiation grafting technique. The silica NPs have been incorporated into the PVDF matrix by a simple melt-blending. Figure 7 shows the SEM morphologies of the silica NPs, which have different surface modifications and content, blending with PVDF matrix. It is observed that both the pristine SiO_2_ and SiO_2_-vinyl shown a significantly agglomeration in the PVDF matrix results in severe phase separation in the nanocomposites (Figure 7a,b). However, the modification of SiO_2_ by Gamma-radiation (F–SiO_2_) can remarkably improve the dispersibility of SiO_2_ particles in the nanocomposites (Figure 7c). To further differentiate the component of nanoparticles in the PVDF phase, the EDX and AFM was used to analyse the different parts of the nanocomposite [31,32,33], as shown in Figures S3 and S4. It was similar to the result of SEM where only the F–SiO_2_ nanoparticles can be uniformly dispersed in the PVDF matrix. We proposed that it was directly related to the immobilization effect of PVDF onto the solid inorganic core. Pristine SiO_2_ and SiO_2_-vinyl have a highly specific surface energy with the PVDF matrix due to the weak interaction by the inherent propensity. However, grafting of PVDF onto the SiO_2_ surface greatly improved the compatibilization between the NPs and polymer matrix, leading to the SiO_2_ particles dispersed in the PVDF uniformly.

The surface modification silica by the radiation grafting strategy can also improve the nanoparticle content in the PVDF matrix. When increasing the indicated amount of silica NPs from 3 wt% to 35 wt%, the aggregation of pristine SiO_2_ in the SiO_2_/PVDF samples was significantly enhanced (Figure S5). The macroscopic phase separation could be observed when the filling amount of silica exceeds 20 wt% (Figure S6), indicating the immiscible between silica with the PVDF matrix. However, for the addition of F–SiO_2_, the dispersion of nanoparticles was a remarkable improvement and the filling rate could reach 35 wt% without phase separation (Figure 7d–f). This again means that the coating of PVDF on silica enhanced the compatibilization of nanoparticles with the PVDF matrix and the radiation grafting was an effective strategy to surface modification PVDF on silica nanoparticles.

### 3.4. Modification Silica Loading Effect on the Physical Properties of the PVDF Matrix

#### 3.4.1. Mechanical Properties

**Figure 8:**
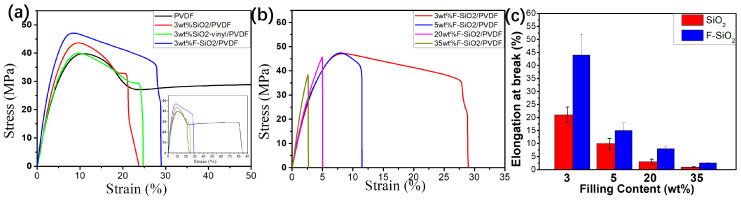
(**a**) Stress-strain curves of the PVDF matrix incorporated with 3 wt% SiO_2_, SiO_2_-vinyl, and F-SiO_2_ nanoparticles, respectively. (**b**) Stress-strain curves of the PVDF matrix incorporated with 3 wt%, 5 wt%, 20 wt%, and 35 wt% F-SiO_2_ nanoparticles, respectively. (**c**) The elongation at break of the F–SiO_2_/PVDF with the addition of a different content of pristine SiO_2_ and F–SiO_2_, respectively.

Tensile tests were carried out on an Instron universal material testing instrument at 25 °C with a tensile speed of 5 mm/min. The corresponding tensile properties of PVDF filling with the different content of pristine silica and F-SiO_2_ were shown in Figure 8, respectively. Neat PVDF shown a typical low yield strength (41.6 ± 1.4 MPa) and low modulus (782 ± 80 MPa) tensile behavior due to the low surface energy of the fluorine element in the main chain. With 3 wt% amount of pristine silica, both the yield strength (43.2 ± 0.8 MPa) and modulus (877 ± 118 MPa) of PVDF nanocomposites get enhanced. However, the improvement of the mechanical performance remained at a low efficiency. Similarly, the addition of SiO_2_-vinyl did not improve either the yield strength or the modulus. The mechanical property was even lower than that of the pristine SiO_2_ samples. These results were due to the bad dispersion properties and poor compatibility of silica with the PVDF matrix. On the other hand, the composites incorporation of nanoparticles in which grafting of PVDF onto the silica surface underwent an observable enhancement in mechanical strength compared with the same content of pristine silica. The yield strength and modulus of 3 wt% F-SiO_2_/PVDF are 45.8 ± 1.0 MPa and 1024 ± 40 MPa, respectively. In addition, both the yield strength and modulus of the F-SiO_2_/PVDF can be significantly improved, and the decrease of the elongation at break by the inherent propensity of SiO_2_ was suppressed with the increase of F–SiO_2_ content (Figure 8b,c). Thus, we were reasonable to deduce that the self-entanglements of a grafted PVDF chain on the SiO_2_ surface with the PVDF matrix reinforced the interactions between the nanoparticles and polymer, which accounted for the improvement of yield strength and modulus in nanocomposites.

#### 3.4.2. The Crystallization Behavior

**Figure 9:**
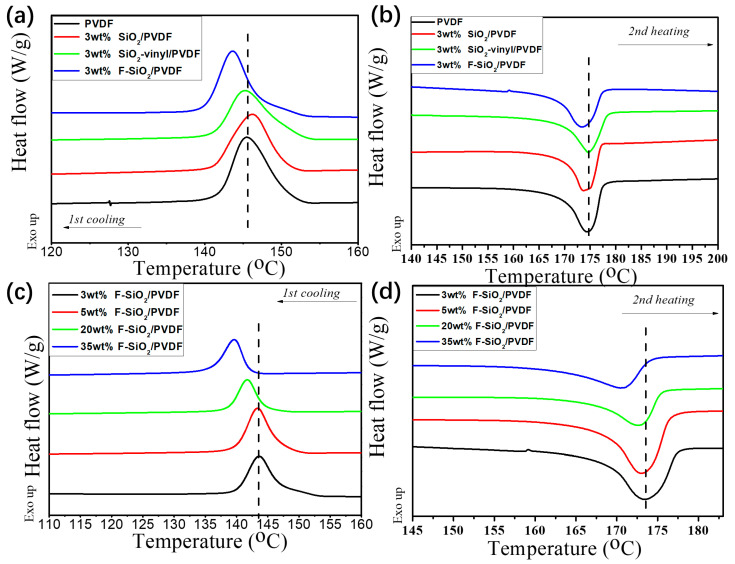
DSC spectra of 1st cooling (**a**) and 2nd heating (**b**) of the PVDF matrix incorporated with 3 wt% SiO_2_, SiO_2_-vinyl, and F-SiO_2_ nanoparticles, respectively. DSC spectra of first cooling (**c**) and second heating (**d**) of the PVDF matrix incorporated with 3 wt%, 5 wt%, 20 wt%, and 35 wt% F-SiO_2_ nanoparticles, respectively.

The crystallization behavior had strong affinities to the mechanical properties of materials [34,35]. The influence of surface modification silica nanoparticles contents on the crystallization behavior of PVDF in the matrix was examined in Figure 9. It was clear that PVDF exhibited strong crystallization ability with high crystallization temperature (Tc) and narrow half-peak breadth as a semi-crystalline polymer. For pristine SiO_2_ and SiO_2_-vinyl, the crystallization and melting behaviors did not vary significantly in the PVDF matrix. However, the melt (T_m_) and crystallization (T_c_) temperature of F-SiO_2_/PVDF nanocomposites were clearly obstructed (Figure 9a,b). This was because the grafted PVDF on the SiO_2_ surface have a strong interaction with the PVDF matrix, which broke the regularity of the molecular chain in the PVDF matrix and increased the hindrance during migration and arrangement when cooling down from the melt.

On the other hand, the change of nanoparticle content in the PVDF matrix can also impact the crystallization behavior of the nanocomposites. For pristine SiO_2_, the crystallization and melting behaviours did not vary significantly in PVDF/SiO_2_ nanocomposites with the change of NPs’ filling content (Figure S7). However, the higher content of F–SiO_2_ was, the lower T_m_ and T_c_ of nanocomposites became. The half-peak breadth for F–SiO_2_/PVDF samples were also broadened by the increasing addition of F–SiO_2_ (Figure 9c,d). It should be noted that the relative location of the PVDF diffraction peak did not change and show a significant structure of PVDF-αphases with various modification nanoparticles addition (Figure S8). Thus, we ascribed the improvements in the mechanical properties of the nanocomposites, which originate from the addition of F–SiO_2_, leading a strong interaction between the F–SiO_2_ surface PVDF coating with the PVDF matrix.

#### 3.4.3. The Thermomechanical Behavior

**Figure 10:**
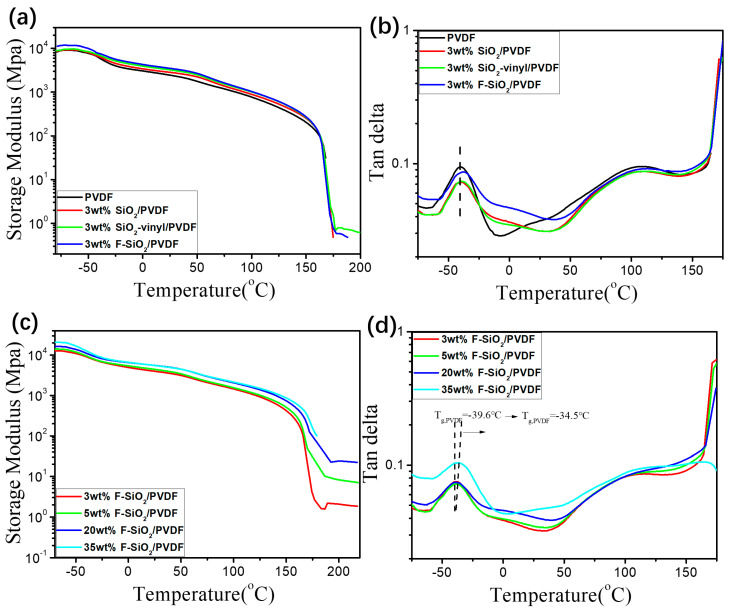
Storage modulus (**a**) and Loss tangent (**b**) as a function of temperature with a frequency of 5 Hz for the PVDF matrix with 3 wt% of SiO_2_, SiO_2_-vinyl, and F-SiO_2_, respectively. Storage modulus (**c**) and Loss tangent (**d**) as a function of temperature with a frequency of 5 Hz for the PVDF matrix with 3 wt%, 5 wt%, 20 wt%, and 35 wt% of F-SiO_2_, respectively. These results were obtained by dynamic thermomechanical analysis (DMA).

Generally speaking, the compatibilization of nanoparticles with polymers determined the glass transition behaviors of nanocomposites and can be observed with the changing of thermomechanical properties [36,37]. To get further information on the effective modification of nanoparticles by radiation grafting, thermomechanical properties of SiO_2_/PVDF nanocomposites are studied by DMA analysis. Figure 10a,b show the storage modulus and loss factor (tanδ) of PVDF nanocomposites with various modified SiO_2_. It can be seen that, with the degree of the surface modification of SiO_2_, the storage modulus of the SiO_2_/PVDF blends increased gradually (Figure 10a). However, the nanocomposites, which have 3 wt% pristine SiO_2_ and SiO_2_-vinyl content nanoparticles, only had the same distinct mechanical relaxation (glass transition temperature, *T_g_*) with the neat PVDF at −42.0 °C. The PVDF incorporation with 3 wt% F-SiO_2_ shows a higher *T_g_* at −39.0 °C (Figure 10b). In addition, with the increase of the filling amount (from 3 wt% to 35 wt%), the *T_g_* and storage modulus of F-SiO_2_/PVDF was further improved, which can be contributed to the enhancement of physical network density and entangled state by limiting the movement of molecular chains in the PVDF matrix (Figure 10c,d). These results are in good accordance with the previous analysis. The increase in DMA indicates that there are strong interactions between F–SiO_2_ and the PVDF matrix, leading to the amelioration in the mechanical properties and dispersity of silica NPs in the PVDF matrix.

#### 3.4.4. The Rheological Properties

**Figure 11:**
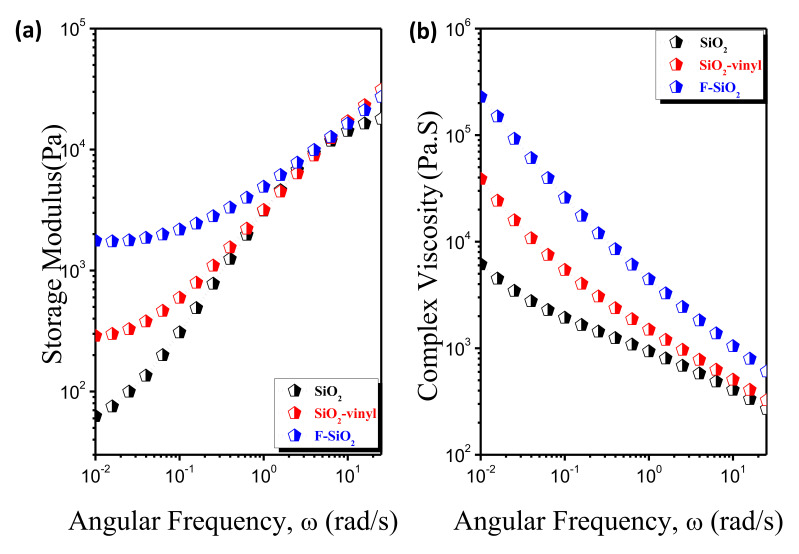
The storage modulus (**a**) and complex viscosity (**b**) of the PVDF matrix incorporated with 3 wt% pristine SiO_2_, SiO_2_-vinyl, and F-SiO_2_ nanoparticles, respectively.

The dynamic rheological response is believed to be an effective method for providing information regarding the structure/morphology of materials under small amplitude oscillatory shear (SAOS). The rheological behaviors of the PVDF matrix with different surface modification SiO_2_ was shown in Figure 11. It is clear that F–SiO_2_ exhibited a completely different response of viscosity and elasticity in the SAOS tests, as compared with the pristine SiO_2_ and SiO_2_-vinyl, which show a sensitive frequency-independent and pseudo solid-like responses at the terminal region. It can be inferred that the self-entanglements of grafted PVDF chain on the SiO_2_ surface with the PVDF matrix reinforced the interactions between nanoparticles and polymer, leading to the effective dispersal in the PVDF matrix and enhancement of interfacial adhesion.

## 4. Conclusions

We demonstrated a facile strategy to graft the polymer onto the surface of nanoparticles through a radiation technique. SiO_2_ NPs were vinyl functionalized (SiO_2_-vinyl) by using γ-methacryloxy propyl-trimethoxylsilane. The SiO_2_-vinyl were then physically mixed with PVDF, which was followed by radiation from the Co^60^ gamma ray at room temperature. PVDF radicals were generated directly and then chemically grafted onto the surface of SiO_2_ nanoparticles by the linking of the double bond on the NPs. The graft ratio of PVDF on SiO_2_ NPs surface can be simply controlled by adjusting the absorbed dose and feed ratio of PVDF to SiO_2_ NPs. The prepared F–SiO_2_ were then dispersed in the PVDF matrix to make the nanocomposites. It was found that the F–SiO_2_ dispersed uniformly in the PVDF matrix, as compared with SiO_2_ and SiO_2_-vinyl. Besides, the filling content of F–SiO_2_ in the PVDF matrix is almost two times higher than the pristine SiO_2_ counterpart. Accordingly, the mechanical property of the F-SiO_2_/PVDF nanocomposites is significantly improved. Therefore, it is a promising way to combine the nanoparticles with polymers efficiently using a simple radiation-induced grafting method. Considering the increasing demand for the hybrid nanoparticles, this approach could be applied to various combinations of nanoparticles and polymers, providing new possibilities for surface modification nanoparticles in industrial production.

## Supplementary Materials

The following are available online at https://www.mdpi.com/2079-4991/10/11/2237/s1. Formula S1: Formula for calculating the grafting ratio of PVDF on F-SiO_2_ nanoparticles surface. Figure S1: ^13^C-NMR spectra of pristine SiO_2_, SiO_2_-vinyl and F-SiO_2_ nanoparticles, respectively. Figure S2: (a) XRD patterns of pristine SiO_2_, SiO_2_-vinyl, F-SiO_2_, and PVDF. (b) Enlarged XRD patterns of pristine SiO_2_, SiO_2_-vinyl, F-SiO_2_ in the 2θ range of 15–30°. (c) Enlarged Rietveld refinement of the pristine SiO_2_, SiO_2_-vinyl, and F-SiO_2_ XRD data in the 2θ range of 15–30°. Figure S3: SEM and Elemental Mapping image (EMI) of PVDF matrix incorporated with 3 wt% (a) pristine SiO_2_, (b) SiO_2_-vinyl, and (c) F-SiO_2_ nanoparticles, respectively. The subscript of 1 and 2 correspond to the signal of silicon and fluoride element in EMI, respectively. Figure S4: AFM of PVDF matrix incorporated with three wt% (a) pristine SiO_2_, (b) SiO_2_-vinyl, and (c) F-SiO_2_ nanoparticles, respectively. Figure S5: SEM image of PVDF matrix incorporated 5 wt% (a), 20wt% (b), 35 wt%, and (c) SiO_2_ nanoparticles, respectively. Figure S6: Digital photograph of the PVDF matrix incorporated 3 wt% (a)—35 wt% (g) SiO_2_ nanoparticles, respectively. Digital photograph of the PVDF matrix incorporated 3 wt% (h)—35 wt% (n) F-SiO_2_ nanoparticles, respectively. Figure S7: DSC spectra of 1st cooling (c) and 2nd heating (d) of the PVDF matrix incorporated with 3 wt%, 5 wt%, 20 wt%, and 35 wt% SiO_2_ nanoparticles, respectively. Figure S8: XRD spectra of the PVDF matrix incorporated with 3 wt% SiO_2_, SiO_2_-vinyl, and F-SiO_2_ nanoparticles, respectively. Table S1: Molecular parameter of the surface modified silica NPs. Table S2: The graft content of PVDF grafted onto silica nanoparticles under a different reactant ratio (wt%/wt%) and radiation dose.
