# Supplementary material for: Radiation Induced Surface Modification of Nanoparticles and Their Dispersion in the Polymer Matrix
Source: Nanomaterials (Basel). 2020 Nov 11;10(11):2237. doi: 10.3390/nano10112237 (PMC7697188; doi:10.3390/nano10112237)
Supplement: Supplementary file 1 [file nanomaterials-10-02237-s001.pdf]

# Supplementary Materials: Radiation Induced Surface Modification of Nanoparticles and their Dispersion in Polymer Matrix

**Zhiang Fu [1,2,3], Xiaoying Gu [2], Lingmin Hu [2], Yongjin Li [2,*] and Jingye Li [1,*]**

**Table of contents**



| | |
|---|---|
| **Equation S1** | Equation for calculating the grafting content of PVDF on F-$SiO_2$ nanoparticles surface. |
| **Figure S1** | $^{13}$C-NMR spectra of pristine $SiO_2$, $SiO_2$-vinyl and F-$SiO_2$ nanoparticles, respectively. |
| **Figure S2** | (a) XRD patterns of pristine $SiO_2$, $SiO_2$-vinyl, F-$SiO_2$ and PVDF; (b) Enlarged XRD patterns of pristine $SiO_2$, $SiO_2$-vinyl, F-$SiO_2$ in the 2θ range of 15-30°. (c) Enlarged Rietveld refinement of the pristine $SiO_2$, $SiO_2$-vinyl and F-$SiO_2$ XRD data in the 2θ range of 15-30°. |
| **Figure S3** | SEM and Elemental Mapping image (EMI) of PVDF matrix incorporated with 3wt% (a) pristine $SiO_2$, (b) $SiO_2$-vinyl and (c) F-$SiO_2$ nanoparticles, respectively; the subscript of 1 and 2 are correspond to the signal of silicon and fluoride element in EMI, respectively. |
| **Figure S4** | AFM of PVDF matrix incorporated with 3wt% (a) pristine $SiO_2$, (b) $SiO_2$-vinyl and (c) F-$SiO_2$ nanoparticles, respectively. |
| **Figure S5** | SEM image of PVDF matrix incorporated 5wt%(a), 20wt%(b), 35wt%(c) $SiO_2$ nanoparticles, respectively. |
| **Figure S6** | Digital photograph of PVDF matrix incorporated 3wt% (a) - 35wt% (g) $SiO_2$ nanoparticles, respectively; Digital photograph of PVDF matrix incorporated 3wt% (h) - 35wt% (n) F-$SiO_2$ nanoparticles, respectively. |
| **Figure S7** | DSC spectra of 1st cooling (c) and 2nd heating (d) of PVDF matrix incorporated with 3wt%, 5wt%, 20wt%, 35wt% $SiO_2$ nanoparticles, respectively. |
| **Figure S8** | XRD spectra of PVDF matrix incorporated with 3 wt% $SiO_2$, $SiO_2$-vinyl and F-$SiO_2$ nanoparticles, respectively. |
| **Table S1** | Molecular parameter of the surface modified silica NPs |
| **Table S2** | The graft content of PVDF grafted onto silica nanoparticles under different reactant ratio (wt% /wt%) and irradiation dose |

$$w_{\text{PVDF}} = \frac{m_{graft\ PVDF}}{m_{F-SiO_2}} = \frac{f_{SiO_2}\cdot(f_{SiO_2-vinyl} - f_{F-SiO_2})}{f_{SiO_2}\cdot f_{SiO_2-vinyl}\cdot(f_{F-SiO_2} - f_{PVDF}) + f_{SiO_2}\cdot(f_{SiO_2-vinyl} - f_{PVDF}) - f_{SiO_2-vinyl}\cdot(f_{F-SiO_2} - f_{PVDF})} \quad (1)$$

**Equation S1.** Equation for calculating the grafting content of PVDF on F-$SiO_2$ nanoparticles surface. Where $w_{\text{PVDF}}$ is the grafting ratio of the grafted PVDF chain on the surface of $SiO_2$, which based on the TGA data. $m_{graft\ PVDF}$ is the actual number mass of grafting PVDF chain on the surface of $SiO_2$. $m_{F-SiO_2}$ is the number mass of the F-$SiO_2$ nanoparticles, which calculated by the content of PVDF and γ-MPS on the F-$SiO_2$ nanoparticles surface. $f_{SiO_2}$, $f_{SiO_2-vinyl}$, $f_{F-SiO_2}$ and $f_{PVDF}$ were represented the inorganic residual content of pristine $SiO_2$, $SiO_2$-vinyl, F-$SiO_2$ and PVDF after the TGA test respectively.

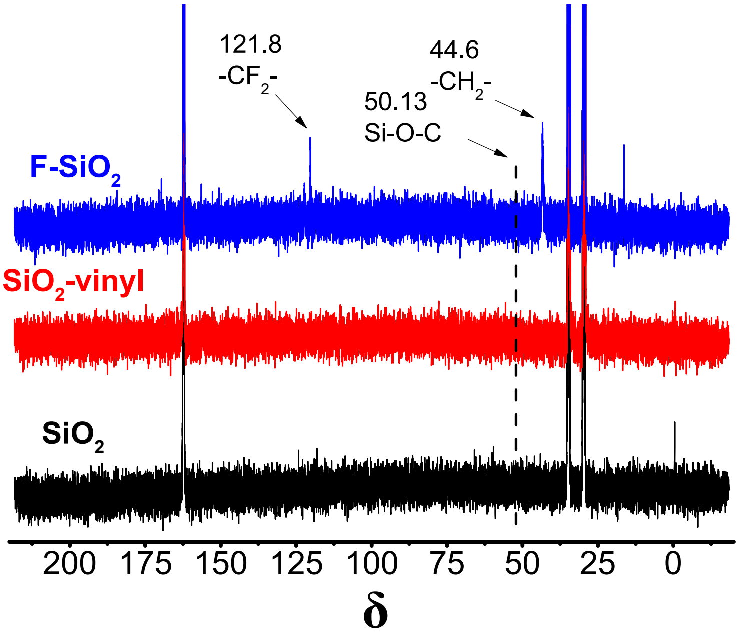


**Figure S1.** $^{13}$C-NMR spectra of pristine $SiO_2$, $SiO_2$-vinyl and F-$SiO_2$ nanoparticles, respectively.

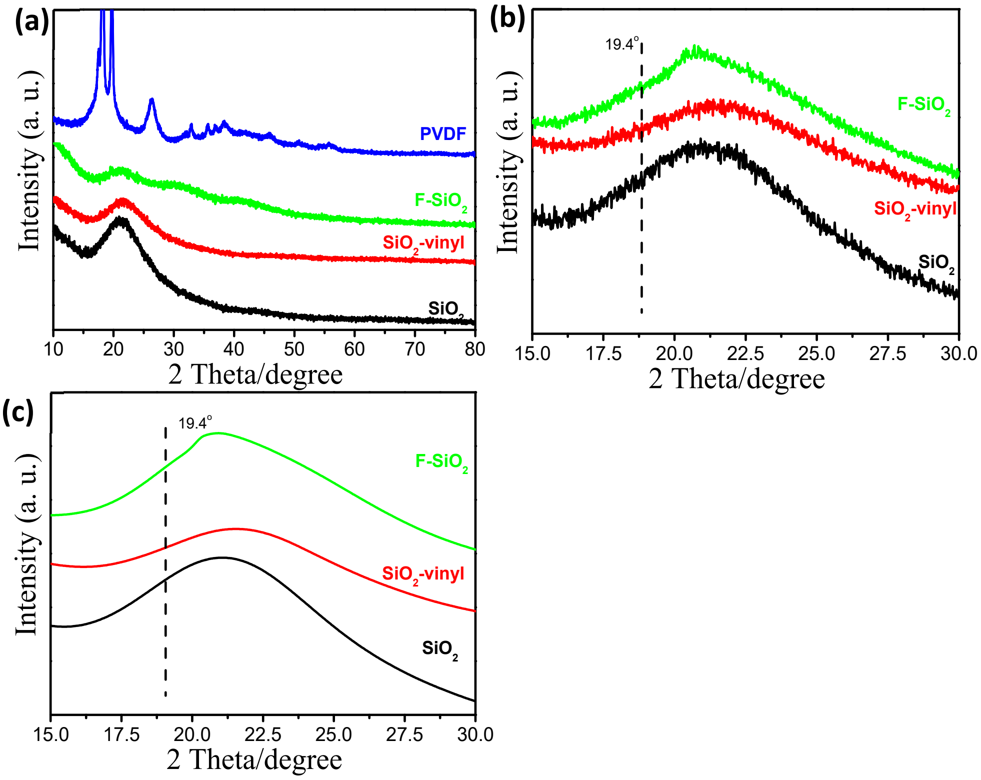


**Figure S2.** (**a**) XRD patterns of pristine $SiO_2$, $SiO_2$-vinyl, F-$SiO_2$ and PVDF; (**b**) Enlarged XRD patterns of pristine $SiO_2$, $SiO_2$-vinyl, F-$SiO_2$ in the 2θ range of 15–30°. (**c**) Enlarged Rietveld refinement of the pristine $SiO_2$, $SiO_2$-vinyl and F-$SiO_2$ XRD data in the 2θ range of 15–30°.

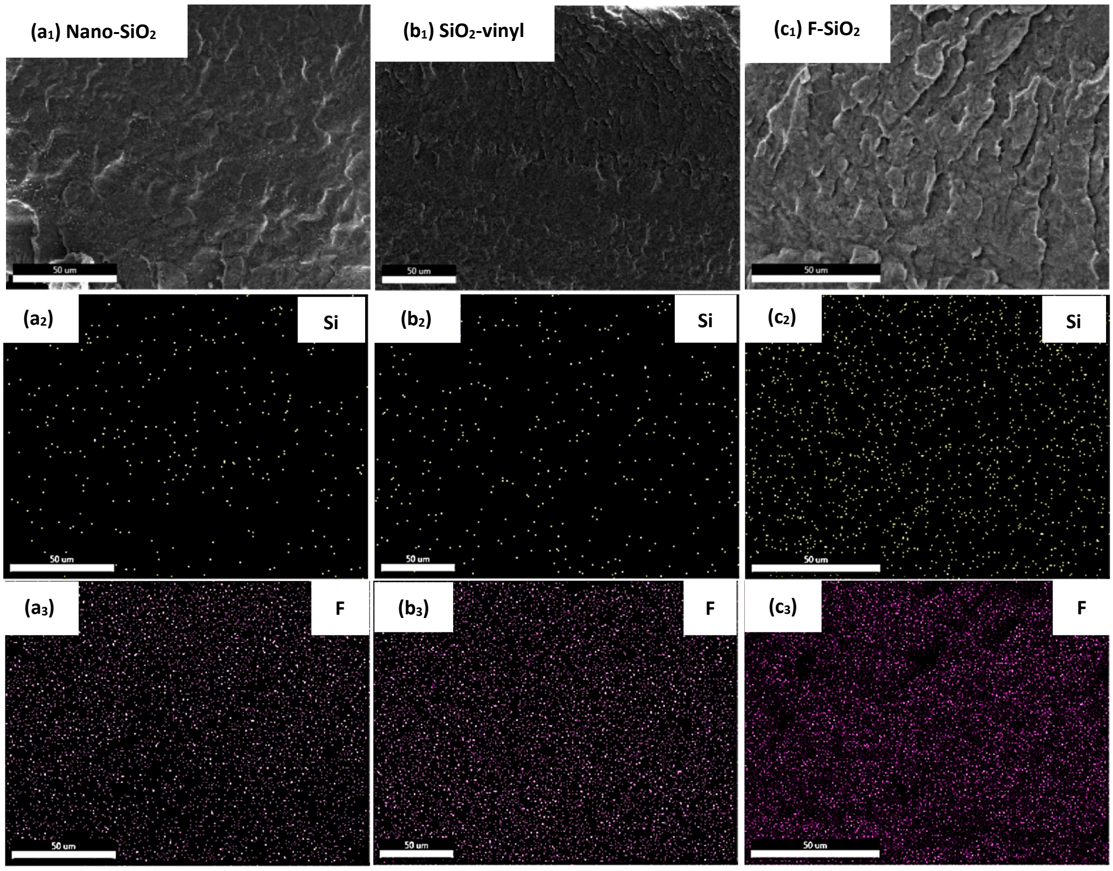

**Figure S3.** SEM and Elemental Mapping image (EMI) of PVDF matrix incorporated with 3wt% (**a**) pristine $SiO_2$, (**b**) $SiO_2$-vinyl and (**c**) F-$SiO_2$ nanoparticles, respectively; the subscript of 1 and 2 are correspond to the signal of silicon and fluoride element in EMI, respectively.

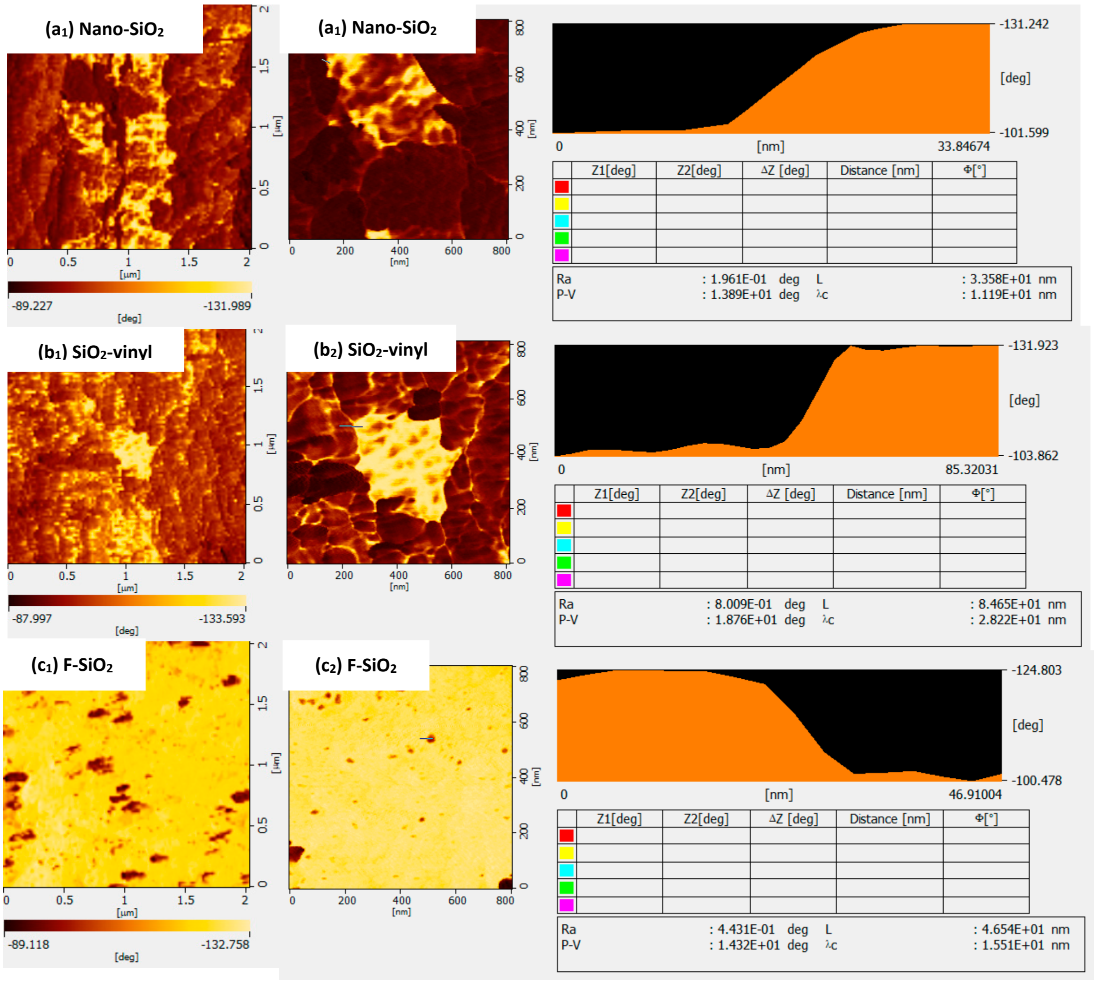


**Figure S4.** AFM of PVDF matrix incorporated with 3wt% (**a**) pristine $SiO_2$, (**b**) $SiO_2$-vinyl and (**c**) F-$SiO_2$ nanoparticles, respectively.

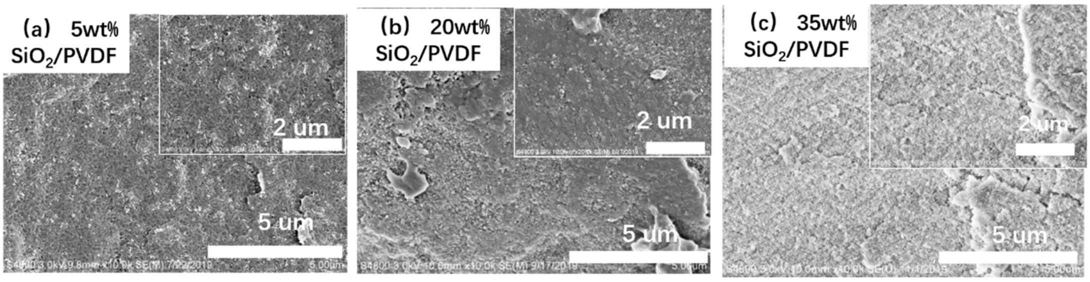


**Figure S5.** SEM image of PVDF matrix incorporated 5wt% (**a**), 20wt% (**b**), 35wt% (**c**) $SiO_2$ nanoparticles, respectively.

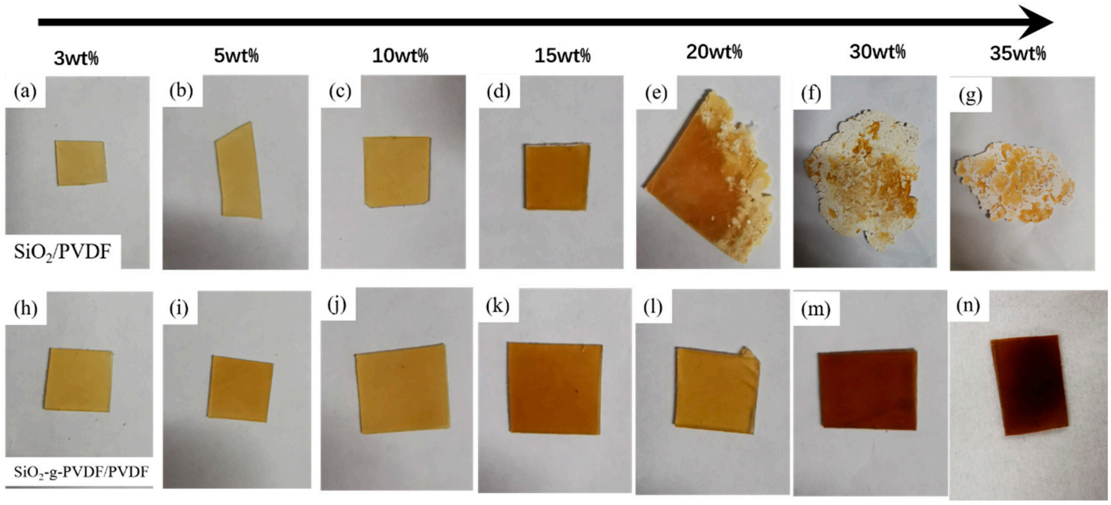


**Figure S6.** Digital photograph of PVDF matrix incorporated 3wt% (**a**–**g**) 35wt% $SiO_2$ nanoparticles, respectively; Digital photograph of PVDF matrix incorporated 3wt% (**h**–**n**) 35wt% F-$SiO_2$ nanoparticles, respectively.

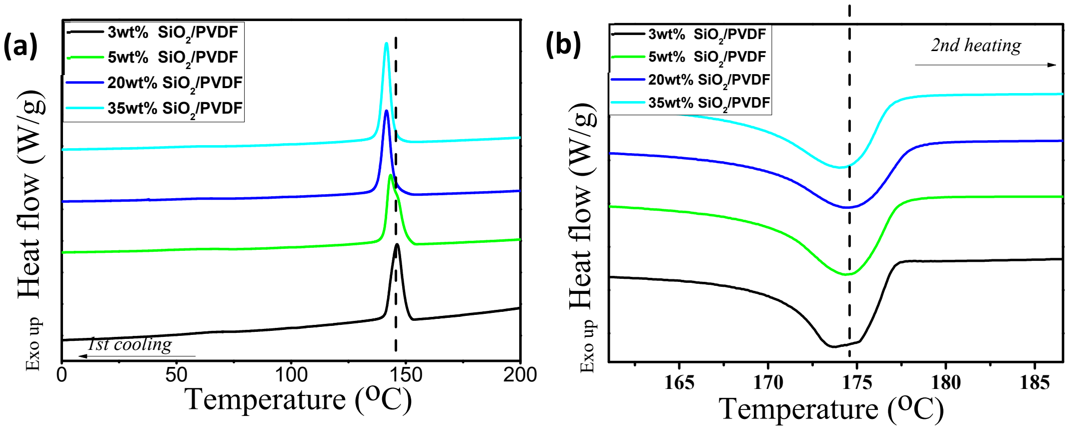


**Figure S7.** DSC spectra of 1st cooling (**a**) and 2nd heating (**b**) of PVDF matrix incorporated with 3wt%, 5wt%, 20wt%, 35wt% $SiO_2$ nanoparticles, respectively.

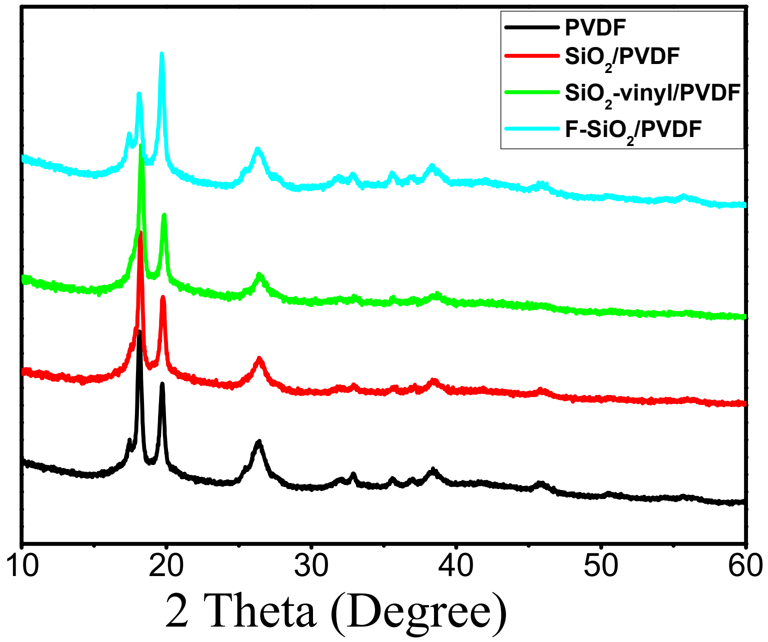


**Figure S8.** XRD spectra of PVDF matrix incorporated with 3 wt% $SiO_2$, $SiO_2$-vinyl and F-$SiO_2$ nanoparticles, respectively.

**Table S1.** Molecular parameter of the surface modified silica NPs.

| **Sample** | ***d*** [a] ***(nm)*** | $T_d$ [b] (°C) | $f_{inorganic}$ [b] (%) | $w_{\gamma-MPS}$ [c] (%) | $w_{PVDF}$ [c] (%) |
|---|---|---|---|---|---|
| $SiO_2$ | 10.3±1.6 | - | 98.0 | - | - |
| $SiO_2$-vinyl | 10.1±2.1 | 439.6 | 95.2 | 3.4 | - |
| F-$SiO_2$ | 15.3±3.5 | 467.4 | 74.5 | 3.4 | 35.9 |

[a] Measured from TEM images (*d*: number averaged diameter of nanoparticles).[b] Measured from the TGA curves of purified specimen ($T_d$: the TGA temperature with maximum loss rate; $f_{inorganic}$: inorganic residual content of modification nanoparticles after TGA test). [c] Calculated by equation (1) based on TGA data ($w_{\gamma-MPS}$: graft content of γ-MPS functioned on the silica nanoparticles surface; $w_{\gamma-MPS}$: graft content of PVDF grafted onto the silica nanoparticles surface;).

**Table S2.** The graft content of PVDF grafted onto silica nanoparticles under different reactant ratio (wt% /wt%) and irradiation dose.

| **Code** | **[SiO2]:[PVDF] (wt% / wt%)** | **Absorbed Dose (kGy)** | **Time (h)** | $T_d$ [a] **(°C)** | $f_{inorganic}$ [b] **(%)** | $w_{PVDF}$ [b] **(%)** |
|---|---|---|---|---|---|---|
| 1 | 1:0.1 | 30 | 17 | 433.3 | 92.7 | 4.0 |
| 2 | 1:0.3 | 30 | 17 | 436.9 | 90.0 | 6.8 |
| 3 | 1:0.5 | 30 | 17 | 438.2 | 88.8 | 8.1 |
| 4 | 1:0.7 | 30 | 17 | 445.7 | 82.0 | 15.1 |
| 5 | 1:1 | 30 | 17 | 460.4 | 79.5 | 17.7 |
| 6 | 1:2 | 30 | 17 | 467.4 | 74.5 | 26.1 |
| 7 | 1:2 | 50 | 17 | 465.7 | 67.0 | 30.6 |
| 8 | 1:2 | 70 | 17 | 458.1 | 66.4 | 31.3 |

[a] Measured from the TGA curves of purified specimen ($T_d$: the TGA temperature with maximum loss rate; $f_{inorganic}$: inorganic residual content of modification nanoparticles after TGA test).[b] Calculated

by equation (1) based on TGA data ($w_{\gamma-MPS}$: graft content of PVDF grafted onto the silica nanoparticles surface).
